# Enhancing anti-AML activity of venetoclax by isoflavone ME-344 through suppression of OXPHOS and/or purine biosynthesis

**DOI:** 10.21203/rs.3.rs-2843025/v1

**Published:** 2023-04-28

**Authors:** Katie H. Hurrish, Yongwei Su, Shraddha Patel, Cassandra L Ramage, Jenna L. Carter, Holly Edwards, Steven A. Buck, Sandra E. Wiley, Maik Hüttemann, Lisa Polin, Juiwanna Kushner, Sijana H. Dzinic, Kathryn White, Xun Bao, Jing Li, Jay Yang, Julie Boerner, Zhanjun Hou, Gheath Al-Atrash, Sergej N Konoplev, Jonathan Busquets, Stefano Tiziani, Larry H. Matherly, Jeffrey W. Taub, Marina Konopleva, Yubin Ge, Natalia Baran

**Affiliations:** 1 Cancer Biology Graduate Program, Wayne State University School of Medicine, Detroit, MI 48201, USA; 2 Department of Oncology, Wayne State University School of Medicine, Detroit, MI 48201, USA; 3 Molecular Therapeutics Program, Barbara Ann Karmanos Cancer Institute, Wayne State University School of Medicine, Detroit, MI 48201, USA; 4 Department of Leukemia, The University of Texas MD Anderson Cancer Center, Houston, TX 77030, USA; 5 MD/PhD Program, Wayne State University School of Medicine, Detroit, MI 48201, USA; 6 Division of Pediatric Hematology/Oncology, Children’s Hospital of Michigan, Detroit, MI 48201, USA; 7 MEI Pharma, Inc., San Diego, CA 92130, USA; 8 Center for Molecular Medicine and Genetics, Wayne State University School of Medicine, Detroit, MI 48201, USA; 9 Department of Stem Cell Transplantation and Cellular Therapy, The University of Texas MD Anderson Cancer Center, Houston, TX 77030, USA; 10 Department of Hematopathology, MD Anderson Cancer Center, The University of Texas, Houston, TX 77030, USA; 11 Department of Nutritional Sciences, The University of Texas at Austin, Austin, TX 78712, USA; 12 Department of Pediatrics, Wayne State University School of Medicine, Detroit, MI 48201, USA

**Keywords:** Acute myeloid leukemia, ME-344, oxidative phosphorylation, purine biosynthesis, venetoclax

## Abstract

Venetoclax (VEN), in combination with low dose cytarabine (AraC) or a hypomethylating agent, is FDA approved to treat acute myeloid leukemia (AML) in patients who are over the age of 75 or cannot tolerate standard chemotherapy. Despite high response rates to these combination therapies, most patients succumb to the disease due to relapse and/or drug resistance, providing an unmet clinical need for novel therapies to improve AML patient survival. ME-344 is a potent isoflavone with demonstrated inhibitory activity toward oxidative phosphorylation (OXPHOS) and clinical activity in solid tumors. Given that OXPHOS inhibition enhances VEN antileukemic activity against AML, we hypothesized that ME-344 could enhance the anti-AML activity of VEN. Here we report that ME-344 synergized with VEN to target AML cell lines and primary patient samples while sparing normal hematopoietic cells. Cooperative suppression of OXPHOS was detected in a subset of AML cell lines and primary patient samples. Metabolomics analysis revealed a significant reduction of purine biosynthesis metabolites by ME-344. Further, lometrexol, an inhibitor of purine biosynthesis, synergistically enhanced VEN-induced apoptosis in AML cell lines. Interestingly, AML cells with acquired resistance to AraC showed significantly increased purine biosynthesis metabolites and sensitivities to ME-344. Furthermore, synergy between ME-344 and VEN was preserved in these AraC-resistant AML cells. These results translated into significantly prolonged survival upon combination of ME-344 and VEN in NSGS mice bearing parental or AraC-resistant MV4-11 leukemia. This study demonstrates that ME-344 enhances VEN antileukemic activity against preclinical models of AML by suppressing OXPHOS and/or purine biosynthesis.

## Introduction

The 5-year overall survival (OS) rate for acute myeloid leukemia (AML) remains low for adults (<30%) and worse for elderly patients (<10%).^1,2^ For AML patients who are ≥75 years of age or cannot tolerate standard chemotherapy, the selective Bcl-2 inhibitor venetoclax (VEN), in combination with either low-dose cytarabine (AraC) or a hypomethylating agent [azacitidine (AZA) or decitabine], was recently approved as a frontline treatment.^3,4^ Although 70% of AML patients receiving this VEN-based low-intensity induction therapy respond, the incidence of relapse remains high.^5^ Additionally, therapeutic options for patients who relapse after receiving VEN-based therapy are very limited, and survival is dismal.^6,7^ Therefore, new therapies are desperately needed to extend duration of response and OS.

The second-generation isoflavone, ME-344, has been reported to inhibit oxidative phosphorylation (OXPHOS) in solid tumors^8,9^ and was tested in Phase I clinical trials (NCT01544322, NCT02806817, NCT02100007). It showed significant antitumor activity after bevacizumab treatment in patients with early HER2-negative breast cancer.^10^ In a basket trial of advanced/metastatic small cell lung, ovarian and cervical cancers, ME-344 was well tolerated with 11 out of 21 evaluable patients having either a partial response (1 patient) or stable disease (10 patients) after 3 cycles of treatment.^11^ In AML, a preclinical study reported that ME-344 was cytotoxic to cell lines and primary patient samples while sparing normal hematopoietic cells.^12^ However, it remains unknown if ME-344 also reduces OXPHOS in AML cells.^12^

Since recent studies suggest that targeting OXPHOS enhances the antileukemic activity of VEN,^13–15^ we aimed in this study to investigate the antileukemic activity of combined ME-344 and VEN against AML cells and the underlying molecular mechanisms.

## Methods

### Reagents

Reagents were purchased from the following vendors: VEN (Venclexta^®^, AbMole Bioscience Inc., Houston, TX); Lometrexol (LMX, LC Laboratories, Woburn, MA); folate-free RPMI1640 (Fisher Scientific, Gibco^™^, Hanover Park, IL); dialyzed fetal bovine serum (FBS, Sigma-Aldrich, St. Louis, MO). ME-344 was provided by MEI Pharma Inc. (San Diego, CA).

### Cell culture

MV4-11, U937, and THP-1 cell lines were purchased from the American Type Culture Collection (Manassas, VA) and the MOLM-13 line from AddexBio (San Diego, CA). Cell lines were cultured as described^16^ and were authenticated at the Karmanos Cancer Institute. Mycoplasma testing was performed monthly.^17^

MV4-11 and U937 cells with acquired resistance to AraC (MV4-11/AraC-R and U937/AraC-R cells) were cultured in the presence of 1.1 µM and 350 nM AraC, respectively.^18,19^ AML cell lines treated with LMX were cultured in folate-free RPMI1640 media containing 10% dialyzed FBS and 100 U/mL penicillin, and 100 μg/mL streptomycin.^20,21^

### Clinical samples

Diagnostic AML blast samples (n=19) and healthy donor bone marrow (BM) samples (n=4) were retrieved from MD Anderson Cancer Center Leukemia sample bank, the Karmanos Cancer Institute Biobanking and Correlative Sciences Core, and the Children’s Hospital of Michigan Leukemia Cell Bank. Written informed consent was provided according to the Declaration of Helsinki. Samples were purified by Ficoll-Hypaque density centrifugation, then cultured in RPMI1640/10% FBS with 2 mM glutamine (Thermo Fisher Scientific). Patient characteristics are shown in the *Online Supplementary Table S1.*

### MTT assays

MTT (3-[4,5-dimethylthiazol-2-yl]−2,5-diphenyltetrazolium bromide; Sigma-Aldrich) assays were performed as described.^19,22^ Primary AML cells were treated with ME-344, VEN, ME-344+VEN or vehicle for 72 hours, followed by MTT analysis. The extent and direction of the antileukemic interactions were determined by isobologram analyses, as described.^22,23^

### Targeted metabolomics

MV4-11 or MV4-11/AraC-R cells were treated with 200 nM ME-344 or vehicle for 8 hours. The cells were collected, washed with PBS, and cell pellets stored at −80°C until analysis. Metabolites were extracted using 80% methanol, as described.^19,24^ Cellular concentrations of metabolites (normalized to cellular proteins) were quantitatively determined using a LC-MS/MS based targeted metabolomics platform.^25^ Data analysis was performed using MetaboAnalyst 4.0 (www.MetaboAnalyst.ca).^19^ Statistical analysis of individual metabolites between treatment groups was performed by an unpaired t-test. An adjusted p-value or false discovery rate (FDR) of <0.05 was considered statistically significant for both the data and pathway analysis.

### Viability and apoptosis analysis by flow cytometry

AML cell lines were treated with vehicle, ME-344, VEN, LMX, ME-344+VEN, or LMX+VEN for up to 48 hours, followed by Annexin V-fluorescein isothiocyanate (FITC)/propidium iodide (PI) (Beckman Coulter; Brea, CA) staining and flow cytometry analyses.^16,26^ Apoptotic events are displayed as mean percentage of Annexin V^+^/PI^−^ (early apoptotic) and Annexin V^+^/PI^+^ (late apoptotic and/or dead) cells. Combination index values (CI) were calculated using CompuSyn software (ComboSyn Inc. Paramus, NJ). CI<1, CI=1, CI>1 indicate synergistic, additive, and antagonistic effects, respectively.

Primary cells, AML and normal human bone marrow cells (BM), were seeded in 24-well plates in triplicate at the density of 300,000–500,000 cells/mL and treated with ME-344 or DMSO (<0.5% (v/v)) for 8 hours, followed by VEN addition, for a total of 48 hours. Cells were then washed twice with Annexin V Binding Buffer (AVBB), stained with 100 µ L of the antibody mix: CD45-APC-Cy7 (H130), CD34-PE-Cy7 (561), CD38-PerCP-Cy5.5 (HB-7) (Biolegend, San Diego, CA), and Annexin V-FITC (Roche Diagnostics, Indianapolis, IN), as described.^27^

### Measurement of mitochondrial membrane potential

AML cell lines were pre-treated with ME-344 for 8 hours, followed by VEN treatment (1 hour). The cells were then stained with 10 µg/mL of JC-1 dye (Thermo Fisher Scientific) in PBS (37°C, 20 minutes), washed, and analyzed by flow cytometry.

### Immunoblotting

Cells were lysed by sonication in 10 mM Tris-Cl, pH 7.0, containing 1% SDS, protease inhibitors, and phosphatase inhibitors (Roche Diagnostics). Whole cell lysates were subjected to SDS-polyacrylamide gel electrophoresis, transferred onto polyvinylidene difluoride membranes (Thermo Fisher Scientific), and immunoblotted using either anti-PARP (Proteintech, Rosemont, IL), -β-actin (Sigma Aldrich), or -cleaved (cf-) caspase3 (Cell Signaling Technologies, Danvers, MA) antibodies. Immunoreactive proteins were visualized using the Odyssey Infrared Imaging System (Li-Cor, Lincoln, NE).

### Seahorse analysis

AML cell lines and freshly isolated primary AML cells were cultured in RPMI1640 media supplemented with 10% FBS without AraC 3 days prior to experiments. On the day of experiments, cells were resuspended in fresh culture media at a density of 2×10^6^ cells/mL, plated in 24-well plates, treated with either DMSO or 200 nM ME-344 for 8 hours, and followed by VEN treatment for 1 hour. Cells were washed twice in PBS and resuspended in 37°C Seahorse Basal Medium supplemented with 1 mM pyruvate, 2 mM glutamine, and 5 mM glucose, pH 7.4. The Mito Stress Test assay was performed as described.^27,28^

### In vivo MV4-11 xenograft models

Parental MV4-11 or MV4-11/AraC-R cells were injected into the tail-veins of immune compromised NSGS mice (Jackson Laboratory, Bar Harbor, ME) (1×10^6^ cells/mouse) and randomized on day 3 into one of several treatment groups (n=5/group). VEN was given by oral gavage (po) (25 mg/kg daily [qd]; 0.1 mL/inj.) and ME-344 (200 mg/kg every third day [q3d]) via intravenous (iv) injection (0.2 mL/inj.). The MV4-11 and MV4-11/AraC-R models were treated with either vehicle, ME-344, VEN, or ME-344+VEN. One arm of the MV4-11/AraC-R model (n=5) received by intraperitoneal injection (ip) Palm-O AraC (P-AraC; 16 mg/kg; q3dx7; 0.5 mL/inj.). VEN and P-AraC were formulated for injection in 3% ethanol (200 proof) and 1% Tween-80 and sterile water (all v/v; USP grade). ME-344 was prepped fresh from 35 mg/mL liquid stock with sterile dH_2_O. Mice were assessed daily for condition and body weight. All treatments were suspended at the onset of leukemia symptoms (hindleg weakness, >5–10% weight loss, ruffled fur). Mice were euthanized when leukemic symptoms progressed (hindleg paralysis, >10–15% weight loss, internal mass >500 mg); with all outcomes confirmed by necropsy. All mice were provided food and water ad libitum, given supportive fluids and supplements as needed, and housed within an Association for Assessment and Accreditation of Laboratory Animal Care International (AAALAC) accredited animal facility with 24/7 veterinary care. *In vivo* experiments were approved by the Institutional Animal Care and Use Committee at Wayne State University.

### Statistical analyses

Unpaired t-tests were used for comparisons between two treatment groups. One-way ANOVA with Bonferroni correction was used for comparisons between four treatment groups. Error bars represent mean ± standard error; the significance level was set at p<0.05. Overall survival was estimated using the Kaplan–Meier method, and statistical analysis was performed using the log-rank test. Precent increase in lifespan was determined using the formula: treated median – control median/control median x 100; median = median day of death due to leukemic progression. All statistical analyses were performed utilizing GraphPad Prism 9.0.

### Data sharing statement

Data reported in this paper will be provided upon request to either Yubin Ge (gey@karmanos.org), Natalia Baran (NBaran@mdanderson.org), or Marina Konopleva (Marina.Konopleva@einsteinmed.edu).

## Results

### ME-344 synergistically enhances the antileukemic activity of VEN against AML

**Figure 1.**
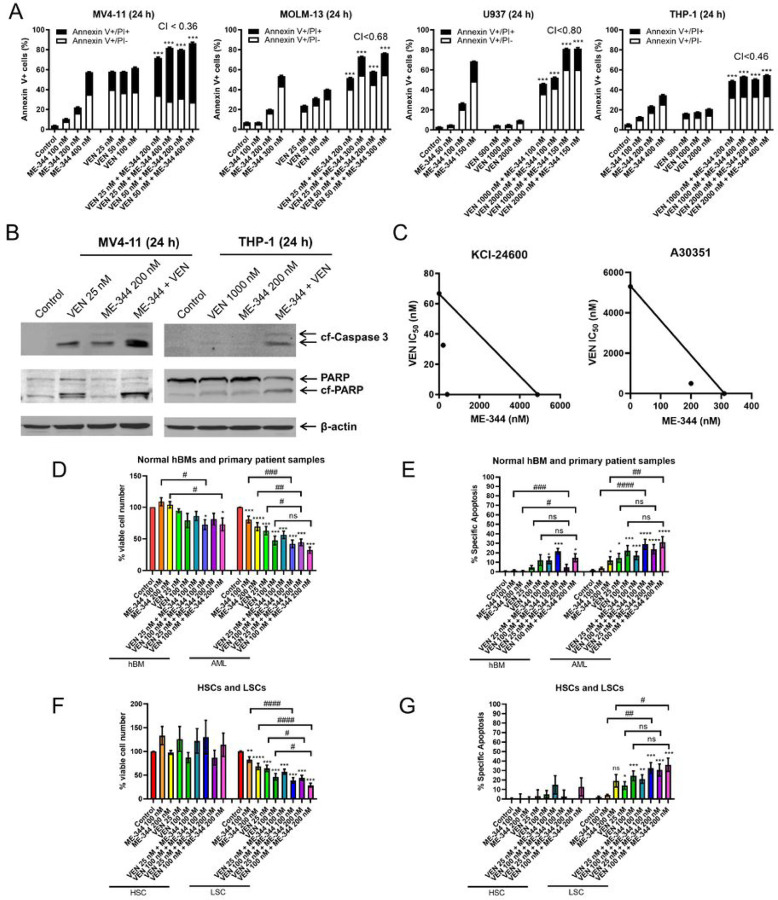
ME-344 synergistically enhances the antileukemic activity of VEN against AML cells. **(A)** AML cell lines were treated with various concentrations of ME-344 and VEN, alone or in combination, or vehicle control for 24 hours. The cells were then stained with Annexin V-FITC/PI and analyzed by flow cytometry. These experiments were performed three times in triplicate; data from one representative experiment is shown. Combination Index (CI) values were calculated using CompuSyn software to determine synergy. CI < 1.0, CI = 1.0, and CI > 1.0 indicate synergistic, additive, and antagonistic effects, respectively. *** indicates p<0.001 compared to vehicle control and single drug treatments. **(B)** Representative western blots of cleaved caspase 3 (cf-Caspase 3) and PARP (cleaved PARP designated as cf-PARP) in MV4-11 and THP-1 cell lines post ME-344 and VEN treatment for 24 hours are shown. Three independent experiments were performed. **(C)** Primary patient samples were treated with ME-344 and VEN in a wide range of concentrations, alone or in combination, for 72 hours and viable cells were determined using the MTT assays. Standard isobologram analysis was performed. The VEN IC_50_ values, in the presence or absence of ME-344, are plotted. The line connecting the single drug IC_50_ values indicates the additive effect, points below the line indicate synergistic effect and those above indicate antagonistic effect. **(D&E)** Human bone marrow cells (hBMs) from healthy donors (n=4) and AML cells from primary patient samples (n=15) were pretreated with ME-344 for 8 hours, followed by VEN treatment for 40 hours, totaling 48 hours. Samples were stained with Annexin V, DAPI, and CD45. Normal hBMs from healthy donors and leukemia cells from primary patient samples were gated by CD45^bright^ and CD45^dim^, respectively. Cell viability was measured by DAPI, while apoptosis was assessed via Annexin V staining. * indicates p<0.05, ** indicates p<0.01, and *** indicates p<0.001 compared to vehicle control. # indicates p<0.05, ## indicates p<0.01, ### indicates p<0.001, and ns indicates not significant for the indicated pairs. (**F&G**) Normal hBMs (panel F, n=4; panel G, n=3) and primary AML patient samples (panel F, n=14; panel G, n=11) were pretreated with ME-344 for 8 hours, followed by VEN for 40 hours, totaling for 48 hours. Then the cells were stained with CD45, CD38, CD34, Annexin V, and DAPI. Normal HSCs and LSCs were gated in the CD45^bright^ and CD45^dim^ population, respectively, as CD38- and CD34+ cells. Cell viability was measured by DAPI, while apoptosis was assessed via Annexin V. * indicates p<0.05, ** indicates p<0.01, and *** indicates p<0.001 compared to vehicle control. # indicates p<0.05, ## indicates p<0.01, ### indicates p<0.001, and ns indicates not significant for the indicated pairs.

To determine whether ME-344 enhances the antileukemic activity of VEN, AML cell lines were treated for 24 hours with ME-344 and VEN, alone or combined, and subjected to apoptosis evaluation. ME-344 induced apoptosis in all AML cell lines tested. VEN also induced apoptosis, except for U937 cells which are intrinsically resistant to VEN.^29^ However upon combinatorial treatment, apoptosis induction was significantly enhanced, demonstrating a synergistic antileukemic interaction (CI<0.8) (Figure 1A), with increased caspase-3- and PARP-cleavage (Figure 1B). To enhance the clinical relevance of our findings, MTT assays were performed with primary naïve AML patient samples treated with ME-344, VEN, or ME-344+VEN for 72 hours. Standard isobologram analyses revealed synergy between ME-344 and VEN in both primary AML patient samples (Figure 1C).

To further support the clinical relevance and estimate therapeutic window, normal human BM samples and primary *de novo* and refractory AML patient samples were treated with ME-344 and VEN for 48 hours and analyzed by flow cytometry. Cells were gated for CD45^bright^ in healthy donor samples and CD45^dim^ in AML patient samples to isolate normal hematopoietic cells and leukemia cells, respectively, from stromal and epithelial cells.^30^ Compared to untreated cells, the viability of CD45^bright^ cells from healthy BM samples was significantly reduced when ME-344 was combined with the highest VEN concentration (100 nM; Figure 1D), though the magnitude was moderate, and apoptosis induction was slightly increased (Figure 1E). In contrast, compared to untreated cells, CD45^dim^ cells from primary AML patient samples had significantly reduced cell viability and increased apoptosis with both single and combination drug treatments, though the combination effects were similar to VEN alone (Figure 1D–E).

Leukemia stem cells (LSCs) are believed to be responsible for relapsed and refractory disease in AML.^1,31,32^ To determine the effects of ME-344 and VEN on normal hematopoietic stem cells (HSCs) and LSCs, normal human BM samples and primary AML patient samples were treated with ME-344 and VEN for 48 hours. The cells were stained with anti-CD45, -CD38, -CD34 antibodies, Annexin V, and DAPI. The CD45^bright^ cells within healthy donor samples and CD45^dim^ population from primary patient samples were further gated for CD38^−^/CD34^+^ cells in the treated and untreated samples.^33^ ME-344 and VEN, both alone and in combination, had no significant effect on normal HSC (CD45^bright^/CD38^−^/CD34^+^) viability (Figure 1F) or apoptosis induction (Figure 1G). In contrast, ME-344 and VEN, both alone and in combination, significantly reduced viable LSCs (CD45^dim^/CD38^−^/CD34^+^) and induced apoptosis, though the combination effects were similar to VEN alone. These results indicate that the combination of ME-344 and VEN targets leukemic cells, both bulk and LSCs, while minimally affecting normal hematopoietic cells, including HSCs.

### ME-344 and VEN cooperate in suppression of OXPHOS in a subset of AML cell lines and primary patient samples

**Figure 2.**
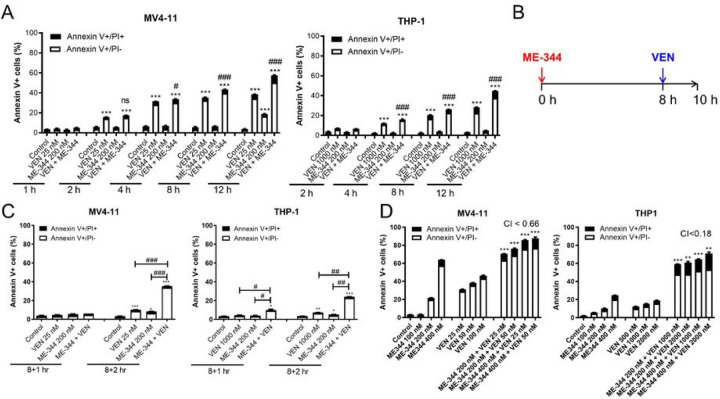
The combination of ME-344 and VEN induces apoptosis in a time-dependent fashion. **(A)** MV4-11 and THP-1 cells were simultaneously treated with ME-344 and VEN for various times, ranging from 1–12 hours. Treated cells were stained with Annexin V-FITC/PI and subjected to flow cytometry analyses. # indicates p<0.05, ## indicates p<0.01, and ### indicates p<0.001 compared to single drug treatments of the same treatment time. * indicates p<0.05, ** indicates p<0.01 and *** indicates p<0.001 compared to vehicle control treated cells of the same treatment time. ns indicates not significant. **(B)** Sequential treatment regimen described for experiments in panel C. **(C)** Annexin V/PI staining and flow cytometry analyses of cells pretreated with ME-344 for 8 hours followed by the addition of VEN for 1 or 2 hours. # indicates p<0.05, ## indicates p<0.01, and ### indicates p<0.001 compared to single drug treatments of the same treatment time. * indicates p<0.05 and *** indicates p<0.001 compared to vehicle control treated cells. **(D)** Annexin V/PI staining and flow cytometry analyses of cells pretreated with ME-344 for 8 hours followed by the addition of VEN for 16 hours, totaling 24 hours of treatment. ** indicates p<0.01 and *** indicates p<0.001 compared to vehicle control and single drug treated cells. Flow experiments shown in panels A, C, and D were performed three times in triplicate; data from one representative experiment is shown.

Preliminary to our mechanistic studies, we designed a sequential treatment schema allowing analysis of drug effects prior to the initiation of apoptosis. ME-344 induced apoptosis in AML cells in 12 hours but enhanced VEN-induced apoptosis in 4 hours, whereas VEN quickly induced apoptosis within 2 hours of exposure in MV4-11 cells and up to 4 hours in THP-1 cells (Figure 2A). Therefore, the sequential treatment involved a pre-treatment with ME-344 (8 hours), followed by VEN treatment for up to 2 hours, totaling up to 10 hours (Figure 2B). VEN and ME-344 cooperatively induced more than 20% apoptosis in both cell lines after 2 hours of VEN treatment, while apoptosis levels post 1 hour of VEN were either at baseline level (in MV4-11) or slightly but significantly higher than baseline level (in THP-1), (Figure 2C). Subsequently, 1 hour of VEN treatment after 8 hours of ME-344 pretreatment was selected for further experiments. To confirm that sequential treatment maintains the drug synergy, MV4-11 and THP-1 cells were pretreated with ME-344 (8 hours) followed by VEN treatment (16 hours), totaling 24 hours. Apoptosis assays and CI calculation confirmed synergistic induction of apoptosis with this sequential treatment schedule (Figure 2D).

**Figure 3.**
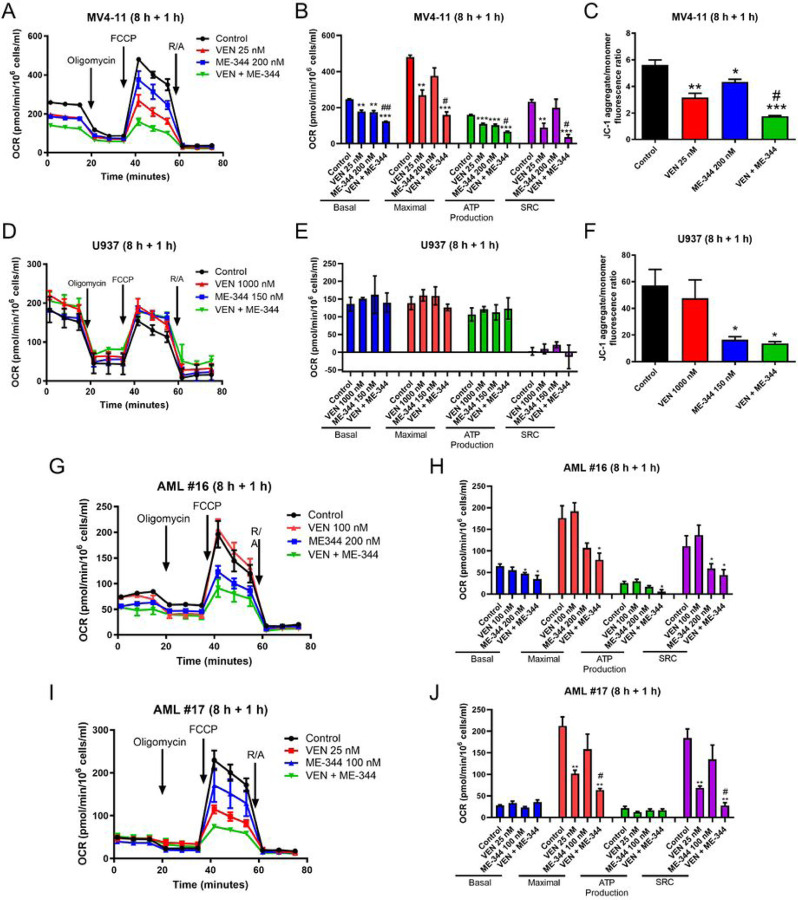
ME-344 and VEN cooperate in suppression of OXPHOS in some AML cells. **(A, B, D, E, G, H, I, and J)** AML cell lines, MV4-11, THP-1, U937, and primary patient samples AML#16 and AML#17 were pre-treated with 200 nM ME-344 for 8 hours, followed by 25 nM VEN for 1 hour. Cells were then subjected to Cell Mito Stress Test. The oxygen consumption rate (OCR) was measured under basal conditions, inhibition of ATP synthase (with 1.5 μM oligomycin), uncoupling of the electron transport chain (ETC) (with 1.0 mM FCCP), and inhibition of complex I and III (with 0.5 μM rotenone and antimycin A (R/A)). All data were normalized to viable cell number at time of assay initiation. Representative OCR plots are shown in panels A, D, G, and I. Basal OCR, maximal OCR, OCR related to ATP production and spare respiratory capacity (SRC) are graphed as mean ± standard errors in panels B, E, H, and J. Statistical significance of the changes was determined with GraphPad Prism 9.0 software. * indicates p<0.05, ** indicates p<0.01 and *** indicates p<0.001 compared to untreated control, while # indicates p<0.05 compared to single drug treatments. **(C&F)** MV4-11 and THP-1 cells were pre-treated with ME-344 for 8 h, followed by VEN treatment for 1 h, for a total of 9 h. Treated and control cells were then stained with JC-1 dye and subjected to flow cytometry analyses to determine the red J-aggregate/green monomer fluorescence ratio, indicative of changes in the mitochondrial membrane potential. # indicates p<0.05 compared to single drug treatments, while * indicates p<0.05, ** indicates p<0.01, and *** indicates p<0.001 compared to vehicle control treated cells.

To determine the mechanism of action of the ME-344 and VEN combination, the XFe96 Seahorse Analyzer was used. Bioenergetic profiles and changes in oxygen consumption rate (OCR) were evaluated via cellular Mito Stress tests in AML cells treated with ME-344 (8 hours), followed by VEN (1 hour). Basal OCR, maximal OCR, OCR related to ATP production, and spare respiratory capacity (SRC) of MV4-11 cells treated with ME-344 and VEN were significantly decreased compared to vehicle control and single drug treatments (Figure 3A&B). JC-1 staining and flow cytometry analysis revealed significant loss of mitochondrial membrane potential (ΔΨm) for single drug treatments compared to control and further significant decrease of ΔΨm for combined ME-344 and VEN treatment compared to individual treatments (Figures 3C and S1). In contrast, U937 cells treated with ME-344 and VEN, alone and in combination, showed no significant effect on OCR (Figure 3D–E). ME-344 treatment, alone and in combination with VEN, showed similar significant decrease of ΔΨm (Figure 3F). In primary AML patient sample AML#16, ME-344 significantly suppressed basal OCR and SRC, along with a borderline significant suppression of maximal OCR (p=0.065) but showed no effect on OCR related to ATP production (Figure 3G–H). Further, its combination with VEN did not cause further suppression of OCR. In primary AML patient sample AML#17, although ME-344 did not show any impact on OCR, it significantly enhanced the suppression of maximal OCR and SRC by VEN (Figure 3I–J).

To determine the importance of OXPHOS suppression for ME-344 and VEN-induced apoptosis, we forced cells to rely on OXPHOS by substituting glucose in culture media with galactose.^34–36^ MV4-11 cells incubated in media supplemented with either glucose or galactose were pretreated with ME-344 (8 hours), followed by the addition of VEN (16 hours; Figure S2A). ME-344-induced apoptosis was enhanced in cells cultured in galactose media compared to glucose media, and further enhanced when used in combination with VEN. While minimal enhancement was detected in THP-1 cells. In contrast, ME-344 and VEN-induced apoptosis (both individually and in combination) was reduced in galactose media-cultured U937 cells. These results demonstrate cooperative suppression of OXPHOS contributes to the mechanism of action in some AML cells and suggest additional mechanisms of action underlying ME-344 and VEN synergy.

### ME-344 decreases purine biosynthesis metabolites and suppression of purine biosynthesis enhances VEN antileukemic activity

**Figure 4.**
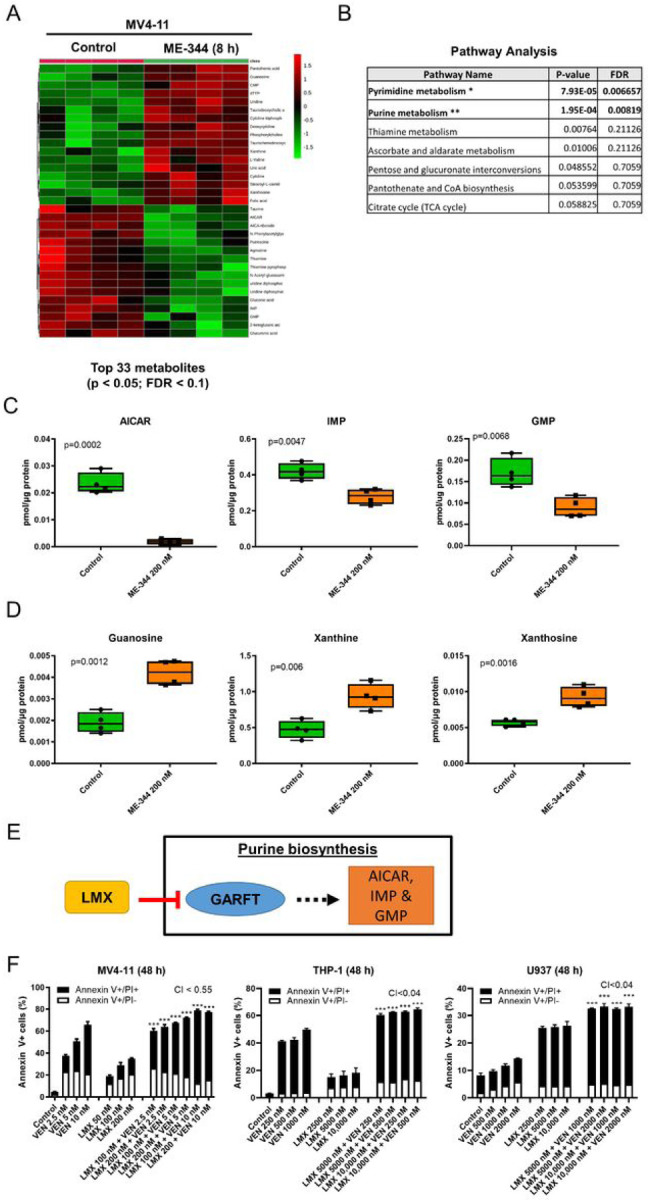
ME-344 suppresses purine biosynthesis and enhances VEN antileukemic activity. **(A-C)** MV4-11 cells were treated with 200 nM ME-344 for 8 hours. Cell pellets were processed and LC-MS/MS detection of approximately 300 cellular metabolites was performed. Levels of metabolites were measured in 4 replicate samples for vehicle control or ME-344 treated cells. Data was entered into the MetaboAnalyst software for statistical analysis. 33 metabolites were determined as significantly altered by ME-344 treatment compared to control cells with p<0.05 and FDR (false discovery rate) <0.1. The data from these 33 metabolites were clustered into a heatmap by the software (panel A). Names of the 33 significantly altered metabolites were logged into the Pathway Analysis tool of MetaboAnalyst. Pathways were ranked based on the impact of each metabolite, and results are shown in panel B. **(C&D)** The levels of metabolites involved in purine biosynthesis pathway (panel C) and salvage pathway (panel D) are graphed as box plots. **(E&F)** MV4-11, THP-1 and U937 cells cultured in folate-free media were treated with variable concentrations of lometrexol alone, VEN alone or in combination, for 48 hours, stained with Annexin V-FITC/PI, and subjected to flow cytometry analyses. These experiments were performed three times in triplicate; data from one representative experiment is shown. Combination Index (CI) values were calculated using CompuSyn software to determine synergy. CI < 1.0, CI = 1.0, and CI > 1.0 indicate synergistic, additive, and antagonistic effects, respectively. *** indicates p<0.001 compared to vehicle control and single drug treatments.

Because studies report ME-344 is a mitochondrial bioenergetics modulator, whether through OXPHOS suppression or additional mechanisms,^8^ we tested the impact of ME-344 on cellular metabolism. A targeted metabolomics analysis of MV4-11 cells revealed that the intracellular concentrations of 33 metabolites were significantly changed by 8 hour ME-344 treatment compared to control (FDR<0.05; designated ME-344 data set) (Figure 4A; Table S2). Pathway analysis using the MetaboAnalyst software identified pyrimidine and purine metabolism as the two significantly affected pathways (Figure 4B). Specifically, metabolites essential for *de novo* purine biosynthesis were significantly decreased, including AICAR, IMP and GMP, while metabolites involved in purine degradation and salvage were increased, including guanosine, xanthine, and xanthosine (Figure 4C–D; Table S2).

Next, we investigated whether suppression of the purine biosynthesis pathway plays a role in the enhancement of VEN activity. AML cells were treated with an inhibitor of glycinamide ribonucleotide formyltransferase (GARFTase), lometrexol ((6R)-DDATHF), which suppresses *de novo* purine biosynthesis upstream of AICAR, IMP and GMP production (Figure 4E),^37^ alone and in combination with VEN. Lometrexol synergistically enhanced the antileukemic activity of VEN in all of the AML cell lines tested (CI<0.6; Figure 4F).

### ME-344 enhances the antileukemic activity of VEN against AraC-resistant AML cells

**Figure 5.**
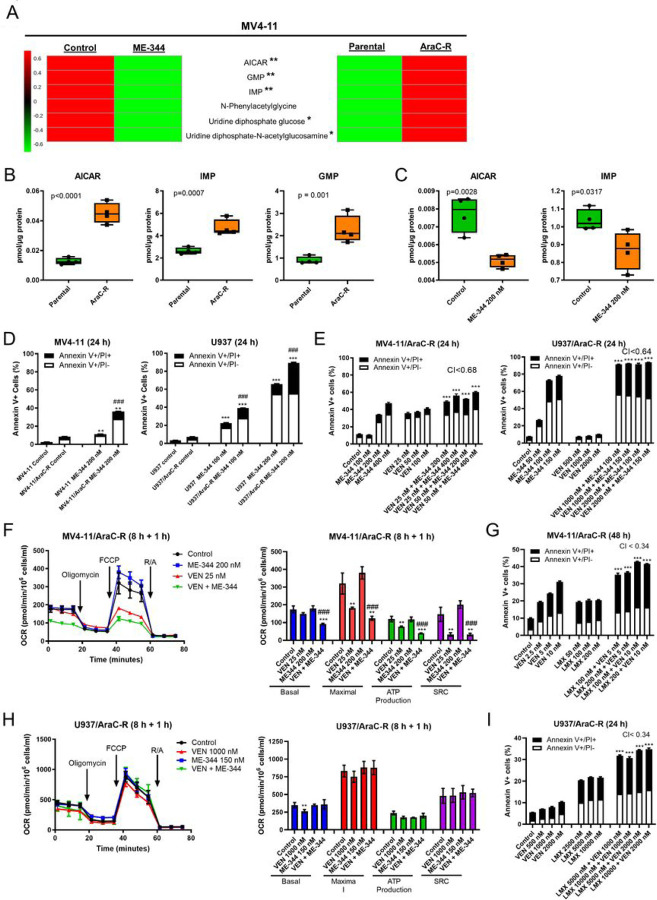
AraC-resistant AML cells show elevated levels of metabolites in the purine biosynthesis pathway and respond to ME-344 and VEN. **(A&B)** LC-MS/MS detection of cellular metabolites was performed in both parental and AraC-resistant MV4-11 cells (MV4-11/AraC-R). Levels of metabolites were measured and analyzed under the same conditions as in Figure 4. 108 metabolites were determined to be significantly altered between parental and MV4-11/AraC-R cells with p<0.05 and FDR <0.1. The metabolomics data set for ME-344 treated vs vehicle control treated was compared to that for MV4-11/AraC-R vs MV4-11. Heatmaps represent the levels of metabolites significantly altered in both datasets with opposite fold change directions. The levels of metabolites involved in purine biosynthesis significantly different in the MV4-11/AraC-R compared to MV4-11 cells are graphed as box plots (panel B). **(C)** MV4-11/AraC-R cells were treated with 200 nM ME-344 for 8 hours. Cell pellets were processed and LC-MS/MS detection of AICAR and IMP was performed. Levels of metabolites were measured in 4 replicate samples for vehicle control or ME-344 treated cells. The levels of metabolites are graphed as box plots. **(D)** To measure apoptosis, MV4-11, MV4-11/AraC-R, U937, and U937/AraC-R cells were treated with ME-344 at the indicated concentrations for 24 hours, stained with Annexin V-FITC/PI and subjected to flow cytometry analyses. These experiments were performed three times in triplicate; data from one representative experiment is shown. ** indicates p<0.01 and *** indicates p<0.001 compared to untreated control, while ### indicates p<0.001 compared to parental with the same treatment. **(E)** MV4-11/AraC-R and U937/AraC-R cells were treated with variable concentrations of ME-344 or VEN, alone or combined, or vehicle control for 24 hours. The cells were then stained with Annexin V-FITC/PI and subjected to flow cytometry analyses. These experiments were performed three times in triplicate; data from one representative experiment is shown. CI value calculation was performed as described in previous figures. *** indicates p<0.001 compared to vehicle control and single drug treatments. **(F&H)** Oxygen consumption rate (OCR) in MV4-11/AraC-R and U937/AraC-R cells pre-treated with ME-344 for 8 hours followed by VEN for 1 hour, alone or combined, were measured over time using a XFe96 Seahorse Analyzer. Baseline OCR measurements were collected during the programed Mito Stress Test assay (1.5 μM oligomycin, 1.0 mM FCCP, and 0.5 μM antimycin/rotenone). All data were normalized to viable cell number at the time of assay initiation. ** indicates p<0.01 and *** indicates p<0.001 compared to untreated control. ### indicates p<0.001 compared to single drug treatments. **(G&I)** MV4-11/AraC-R and U937/AraC-R cells were treated with variable concentrations of lometrexol alone, VEN alone or in combination, for 48 or 24 hours, then stained with Annexin V-FITC/PI, and subjected to flow cytometry analyses. These experiments were performed three times in triplicate; data from one representative experiment is shown. Combination Index (CI) values were calculated using CompuSyn software to determine synergy. CI < 1.0, CI = 1.0, and CI > 1.0 indicate synergistic, additive, and antagonistic effects, respectively. *** indicates p<0.001 compared to vehicle control and single drug treatments.

Resistance to chemotherapy drugs remains a major cause of treatment failure in AML. Therefore, identifying therapies that can target chemotherapy-resistant AML is critical for the improvement of treatment outcome. AraC is a nucleoside analog, hence acquired resistance to this agent might be associated with altered purine and/or pyrimidine metabolism. Metabolomics analysis of MV4-11 cells with acquired resistance to AraC (MV4-11/AraC-R^18^) and the parental MV4-11 cells revealed 100 differentially expressed metabolites (AraC-R data set), and pathway analysis of the AraC-R data set revealed the purine and pyrimidine metabolism pathways among the top pathways affected.^38^ Comparing the AraC-R data set and the ME-344 data set, we identified 6 overlapping metabolites that were oppositely modulated. These included AICAR, IMP, and GMP (purine biosynthesis) and UDP-Glc and UDP-GlcNAc (pyrimidine metabolism).^39,40^ All 6 were upregulated in MV4-11/AraC-R cells compared to parental MV4-11 cells and were downregulated by ME-344 treatment in parental MV4-11 cells (Figure 5A–B; Table S3). Consistent with the parental MV4-11 cells, treatment of MV4-11/AraC-R cells with ME-344 for 8 hours also resulted in significantly decreased levels of AICAR and IMP (Figure 5C). These results suggest that upregulation of *de novo* purine biosynthesis may represent an emerging target in AraC-resistant AML cells, which might be suppressed by ME-344.

To determine whether ME-344 targets AraC-resistant AML cells and enhances VEN activity, parental and AraC-resistant cells were treated with ME-344 (24 hours). ME-344-induced apoptosis was significantly higher in AraC-resistant cells than the parental cells (Figure 5D). Moreover, ME-344 and VEN synergistically induced apoptosis in the AraC-resistant cells (Figure 5E). To investigate whether ME-344 targets OXPHOS when combined with VEN in AraC-resistant cells as Bosc et al reported that AraC-resistant AML cells are more dependent on OXPHOS and are targetable by OXPHOS inhibition,^41^ MV4-11/AraC-R and U937/AraC-R cells were sequentially treated and subjected to respiration evaluation. While ME-344 had minimal effects on OCR in MV4-11/AraC-R cells, it significantly enhanced the suppression of OCR by VEN (Figure 5F). As in the parental MV4-11 cells, inhibition of the *de novo* purine biosynthesis pathway with lometrexol synergistically enhanced apoptosis induced by VEN (Figure 5G). Similar to parental U937 cells, U937/AraC-R cells treated with ME-344 and VEN, alone and in combination, showed no significant changes of OCR (Figure 5H). However, lometrexol and VEN synergistically induced apoptosis in these cells (Figure 5I). These results indicate that cooperative suppression of OXPHOS by both agents and/or suppression of *de novo* purine biosynthesis by ME-344 contributes to the synergy between ME-344 and VEN in AraC-resistant AML cell lines.

### ME-344 combined with VEN prolongs survival of NSGS mice bearing MV4-11 or MV4-11/AraC-R leukemia

ME-344 enhanced the antileukemic activity of VEN *in vitro* against AML cells, including those resistant to AraC. Thus, we evaluated this drug combination in NSGS mice bearing MV4-11 leukemia. We first performed a pilot dose-route optimization study to determine the best route and dose schedule for ME-344. Treatment was initiated on day 4 via either ip or iv administration. Doses were escalated based on mouse body weights and development of symptoms (Figure S3A). Of note, mice in the high dose ip arm had three deaths (day 25, 25, and 28; necropsy determined deaths were due to drug toxicity; LD60), while the lower dose prolonged median survival (MS) = 36 days, mice needed longer recovery time (Figures S3B-C). The iv arm also prolonged survival (37 days) compared to control (31 days; Figure S3D) with no adverse symptoms, so ME-344 at 200 mg/kg (iv) ME-344 was selected as the dose for further studies.

**Figure 6.**
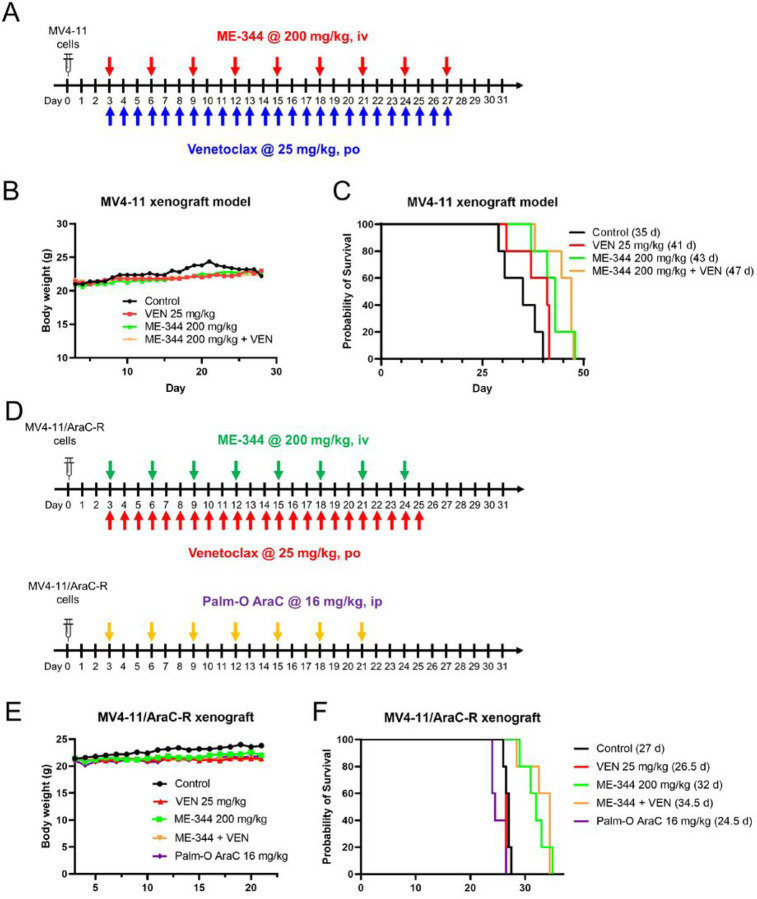
ME-344 alone and its combination with VEN prolong survival of NSGS mice bearing either parental or AraC-resistant MV4-11 leukemia. (A-C) NSGS mice were engrafted with 1×10^6^ MV4-11 cells per mouse through the tail vein (day 0). On day 3, mice were randomized into each treatment arm (n=5), including vehicle control, 200 mg/kg ME-344 (administered intravenously (iv), q3d), 25 mg/kg VEN (oral gavage, qd), or ME-344 + VEN treatment arms (matching single arms). Treatment started on Day 3 and was suspended for all groups on day 28 once onset of leukemic symptoms presented in any mouse. Treatment schema is shown in panel A. Daily average mouse body weights are plotted from day 3 to 28 (panel B). Survival for each treatment arm were estimated using the Kaplan-Meier method and log-rank test for statistical significance (panel C). ME-344 compared to control p=0.0127. ME-344 + VEN compared to control p=0.0088. ME-344 + VEN compared to VEN p=0.0251. ME-344 + VEN compared to ME-344 p =0.7114. (D-F) NSGS mice were engrafted with 1×10^6^ MV4-11/AraC-R cells per mouse, iv via the tail vein and randomized into each treatment arm (n=5), including vehicle control, 200 mg/kg ME-344 (i.v., q3d), 25 mg/kg VEN (oral gavage, qd), ME-344 + VEN treatment arms (matching single arms), or 16 mg/kg Palm-O AraC (i.p., q3d). Treatment started on day 3. Palm-O AraC treatment was stopped early after 7 injections (day 21) due to onset of symptoms in this cohort. ME-344 and VEN, alone and in combination, were suspended at the onset of leukemic symptoms in the control group (day 25). Treatment schemes are shown in panel D. Average mouse body weights (in g) were recorded daily (panel E). Survival for each treatment arm were estimated using the Kaplan-Meier method and log-rank test for statistical significance (panel F). ME-344 compared to control p=0.0021. ME-344 + VEN compared to control p=0.0021. ME-344 + VEN compared to VEN p=0.002. ME-344 + VEN compared to ME-344 p=0.6777.

To evaluate the efficacy of ME-344+VEN combination *in vivo*, mice engrafted with MV4-11 cells were randomized on day 3 to control, VEN (25 mg/kg, qd), ME-344 (200 mg/kg, q3d) or combination treatment groups (Figure 6A). This treatment was carried-out through day 27, when the first signs of leukemia symptoms presented. No weight loss or drug-related toxicities were noted in these therapeutic cohorts (Figures 6B). The ME-344+VEN combination prolonged MS to 47 days, compared to 35 days in the vehicle control arm (p<0.009; Figure 6C). This equates to a 34% ILS (increase in lifespan). ME-344 alone also prolonged lifespan by 23% (MS = 43 days, compared to 35 days in the vehicle control arm, p<0.02). VEN alone produced 17% ILS, indicating cooperative antileukemic activity of ME-344 and VEN against MV4-11 cells *in vivo*. Similar results were obtained with ME-344 at 125 mg/kg (iv; q2d) alone and in combination with 25 mg/kg VEN (qd) (Figure S4).

To determine the antileukemic efficacy of ME-344 and VEN in NSGS mice bearing AraC-resistant MV4-11 leukemia, mice were engrafted (day 0) with MV4-11/AraC-R cells and then randomized on day 3 to vehicle control, VEN (25 mg/kg, qd), ME-344 (200 mg/kg, q3d), P-AraC (16 mg/kg, q3d) or VEN+ME-344 groups (Figure 6D). P-AraC treatment was stopped early, after 7 injections (day 21), due to onset of leukemic symptoms in this cohort (MS = 24.5 days; −9% ILS), confirming these cells are drug resistant. ME-344 and VEN treatments were suspended at onset of leukemia symptoms in the control group (day 25). VEN monotherapy (MS = 26.5 days; −2% ILS) also failed to prolong survival compared to vehicle control (MS = 27 days). However, both ME-344 monotherapy (MS = 32 days; 19% ILS) and ME-344+VEN (MS = 34.5 days; 28% ILS) prolonged median survival. Of note, the combination arm showed efficacy over ME-344- or VEN-alone arm without adverse symptoms or body weight loss (Figure 6E–F). Overall, these results show the ME-344 and VEN combination has *in vivo* efficacy against AML cells, including those with acquired resistance to AraC.

## Discussion

Here, we investigated the antileukemic efficacy of ME-344 in combination with VEN against AML cells both *in vitro* and *in vivo*. ME-344 has been reported to suppress OXPHOS,^9,13,42,43^ and OXPHOS inhibition has been shown to enhance VEN activity against AML cells.^13–15^ We found cooperative suppression of OXPHOS by these two agents in MV4-11 and THP-1 cell lines and one primary AML patient sample (AML#17), but not in U937 AML cell line and the other patient sample (AML#16). This highlights the existence of additional mechanisms that contribute to the enhanced antileukemic activity of VEN by ME-344.

The targeted metabolomics analyses revealed differential expression of metabolites involved in purine metabolism. In AML cells, ME-344 treatment decreased intracellular levels of AICAR, IMP and GMP, indicating suppression of de novo purine biosynthesis. Additionally, ME-344 treatment increased intracellular levels of guanosine, xanthine, and xanthosine (Figure 4), indicating a potential compensatory increase in purine salvage. Lometrexol also synergistically enhanced apoptosis induced by VEN, confirming that suppression of *de novo* purine biosynthesis enhances the antileukemic activity of VEN against AML cells. MV4-11 cells with acquired resistance to AraC showed significantly increased intracellular levels of AICAR, IMP and GMP compared to parental cells. These results are consistent with a recent study reporting that several metabolites in the purine metabolic pathway were differentially expressed between inherently AraC sensitive and resistant AML cell lines.^44^ Taken together, our findings suggests that suppression of *de novo* purine biosynthesis is an additional mechanism of action of ME-344 against AML cells.

Recently, Lin et al. used a loss-of-function CRISPR/Cas9 knockout screen to identify metabolic genes capable of sensitizing AML cells to VEN.^45^ Interestingly, they found that inhibition of *de novo* purine and pyrimidine biosynthesis sensitizes AML cells to VEN treatment. Another genome-wide CRISPR/Cas9 screening identified PAICS, an enzyme involved in de novo purine biosynthesis, as a potential target for AML therapy.^46^ Our results further confirmed that inhibition of *de novo* purine biosynthesis enhances the activity of VEN. This is the first time ME-344 has been reported to presumably suppress purine biosynthesis and showed improved efficacy in AML. We suspect that ME-344 may suppress an enzyme in the *de novo* purine biosynthesis pathway upstream of AICAR, which warrants further investigation. Mitochondrial function may also contribute to the suppression of purine biosynthesis, which requires ATP input. Additionally, mitochondria are physically associated with purinosomes, a complex formed by the *de novo* purine biosynthesis enzymes.^47^ However, ME-344 did not significantly alter OCR nor was there cooperative decrease of ΔΨm in U937 cells, yet ME-344 and GARFTase inhibition synergized with VEN, suggesting that the suppression of *de novo* purine biosynthesis was not solely due to suppression of mitochondrial OXPHOS function.

In summary, our findings demonstrate that ME-344 enhanced VEN activity against AML cell lines and primary AML samples, including those resistant to AraC, through suppression of OXPHOS and/or purine biosynthesis. Combined ME-344+VEN had little to no effect on normal HSCs viability and induced minimal apoptosis, suggesting a therapeutic window may exist. Our results support further development of this promising combination strategy, in both frontline AML to enhance duration of response of venetoclax-based therapies, and in the setting of cytarabine resistance.

## Supplementary Material

Supplement 1

## Key Points:

ME-344 enhances venetoclax activity against AML *in vitro* and *in vivo* via suppression of OXPHOS and/or de novo purine biosynthesis.
