# Supplementary material for: Enhancing anti-AML activity of venetoclax by isoflavone ME-344 through suppression of OXPHOS and/or purine biosynthesis
Source: Res Sq. 2023 Apr 28:rs.3.rs-2843025. Preprint. [Version 1] doi: 10.21203/rs.3.rs-2843025/v1 (PMC10168457; doi:10.21203/rs.3.rs-2843025/v1)
Supplement: Supplement 1 [file NIHPPrs2843025v1-supplement-1.pdf]

Supplementary Files

This is a list of supplementary files associated with this preprint. Click to download.

- supplement.pdf
